# Quick and Sensitive UPLC-ESI-MS/MS Method for Simultaneous Estimation of Sofosbuvir and Its Metabolite in Human Plasma

**DOI:** 10.3390/molecules24071302

**Published:** 2019-04-03

**Authors:** Mohammad H. Semreen, Hasan Y. Alniss, Muath K. Mousa, Hassan Y. Aboul-Enein

**Affiliations:** 1 College of Pharmacy, University of Sharjah, P.O. Box 27272, Sharjah, UAE; halniss@sharjah.ac.ae; 2 Sharjah Institute for Medical Research, University of Sharjah, P.O. Box 27272, Sharjah, UAE; 3 Research Institute of Science and Engineering, University of Sharjah, P.O. Box 27272, Sharjah, UAE; mmousa2@sharjah.ac.ae; 4 Pharmaceutical and Medicinal Chemistry Department, Pharmaceutical and Drug Industries Research Division, National Research Centre, P.O. Box 12622, Dokki, Giza, Egypt; haboulenein@yahoo.com

**Keywords:** sofosbuvir, GS331007, plasma, ultra-performance liquid chromatography, tandem mass spectrometry

## Abstract

A simple, fast and highly sensitive RP-UPLC-MS/MS method was developed and validated for the simultaneous determination of sofosbuvir (SR) and its metabolite GS331007 in human plasma using ketotifen as an internal standard (IS). The separation was achieved on Acquity UPLC BEH C_18_ (50 × 2.1 mm, i.d. 1.7 µm, Waters, USA) column using acetonitrile:5 mM ammonium formate:0.1% formic acid (85:15:0.1% *v*/*v*/*v*) as a mobile phase at a flow rate of 0.35 mL/min in an isocratic elution. The Xevo TQD UPLC-MS/MS was operated under the multiple-reaction monitoring mode using positive electrospray ionization. Extraction with dichloromethane was used in the sample preparation. Method validation was performed as per the Food and Drug Administration (FDA) guidelines and the calibration curves of the proposed method were found to be linear in the range of 1–1000 ng/mL for SR and in the range of 10–1500 ng/mL for its metabolite (GS331007) with an elution time of 1.83 min. All validation parameters were within the acceptable range according to the bioanalytical methods validation guidelines. Furthermore, the obtained results of matrix effects indicate that ion suppression or enhancement from human plasma components was negligible under the optimized conditions. The proposed method can be applied in high-throughput analysis required for pharmacokinetic and bioequivalence studies in human samples.

## 1. Introduction

Sofosbuvir (SR) is a highly potent uridine analogue nucleotide prodrug used for the treatment of the hepatitis C virus (HCV) genotypes 1–4 [1,2,3,4,5,6]. SR selectively targets the nonstructural protein 5B (NS5B) polymerase, which plays a vital role in virus replication.

SR can be used alone or in combination with other drugs such as ledipasvir and ribavirin [7]. Compared with the earlier nucleotide analogues, SR provides a higher prognosis rate, reduced duration of therapy and fewer side effects [3,8,9]. SR has therefore become a major component of many HCV treatment regimens and is considered among the essential forms of treatment according to the World Health Organization (WHO). WHO statistics have shown that the hepatitis C virus has infected 130 million people, and that the majority of patients are chronically infected. The extensive use of SR as a drug of choice in the treatment of the hepatitis C virus necessitates the need to develop a rapid bioanalytical method for the routine measurement of SR in biological samples.

Some analytical methods have been published for the estimation of SR and its metabolite, or in combination with other antiviral agents in biological samples [10,11,12]. One of these methods achieved a retention time of 3.5 min and achieved linear ranges of 5.0–2500 μg/L and 25–5000 μg/L for SF and GS331007, respectively. Another reported method used a solid-phase extraction with a gradient elution of two mobile phases: ammonium acetate 5 mM (pH 9.5) and acetonitrile. This method achieved a lower limit of quantification (LLOQ) of 11.7 ng/mL [13]. Another method was reported for the quantification of SR and its active metabolite GS331007 using multiple reaction monitoring (MRM) and isocratic elution. This method showed an LLOQ of 10 ng/mL [14]. Quantification of ribavirin/SR in combination with the GS331007 metabolite in rat plasma was achieved by UPLC-MS/MS with an LLOQ of 10 ng/mL for SR and GS331007 [15,16]. Simultaneous quantification of antiviral agents such as ledipasvir and SR in rat plasma was also carried out using UPLC-MS/MS and showed a retention time of 1.50 and 1.52 min for ledipasvir and SR, respectively, using gradient elution [17]. 

It is clear that there are a limited number of published bioanalytical procedures for the quantification of SR. Furthermore, the spread of the hepatitis C virus, especially in developing countries, necessitates the need to develop a rapid bioanalytical method for the routine analysis of SR in biological matrices. In this work, we describe the development and validation of UPLC-ESI-MS/MS for the simultaneous quantification of SR and its active metabolite GS331007 by applying an efficient liquid-liquid extraction (LLE). Sample preparation is an essential step in bioanalytical procedures, which aims to remove proteins and minimize endogenous interferences before sample analysis. Several solvents were tried and the best extraction efficiency was obtained by using dichloromethane as a solvent, in contrast to the previously reported methods [15,16] that used ethyl acetate as an extraction solvent. The advantage of using dichloromethane was reflected in the sensitivity of the method, which achieved an LLOQ of 1 ng/mL compared to 10 ng for the previously reported method. This is highly important, especially for the quantitative analysis of drugs and their metabolites in biological samples, where they usually exist at very low concentration (conc.), in addition to achieving a short analysis time (2 min). Thus, this method offers advantages over the other reported methods and can therefore be applied in high-throughput investigations employed in bioanalytical protocols.

## 2. Results 

### Chromatographic Conditions and Sample Preparation 

**Figure 1:**
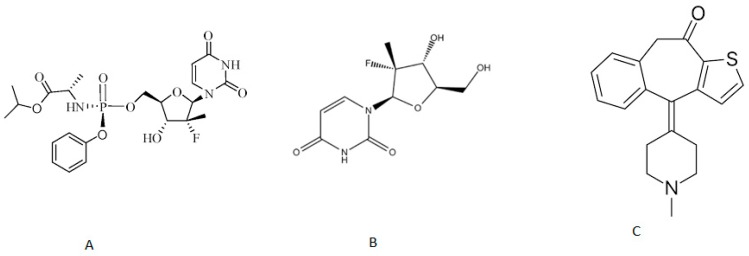
The chemical structures of sofosbuvir (**A**), GS331007 (**B**) and ketotifen (IS) (**C**).

**Figure 2:**
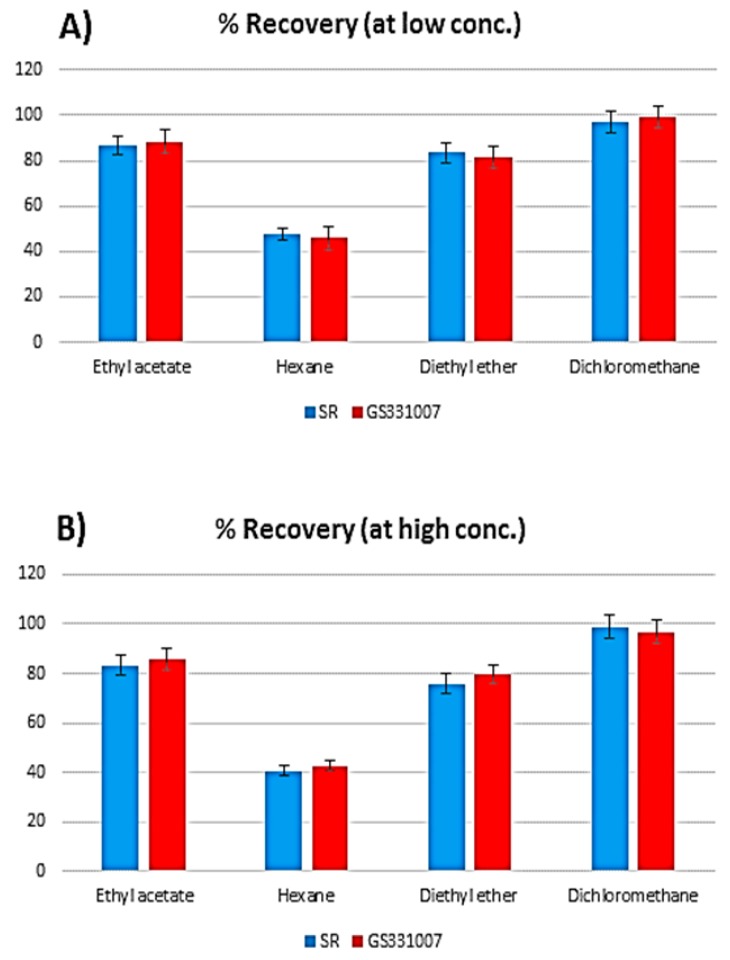
A bar graph showing the extraction solvent effect on the % recoveries of sofosbuvir (SR) and its metabolite GS331007 at low (**A**) and high concentrations (**B**).

In order to extract SR, its metabolite GS331007 and IS (Figure 1) from plasma, different extraction methods were investigated. The LLE efficiency was evaluated using a range of organic solvents with different polarities such as n-hexane, diethyl ether, ethyl acetate and dichloromethane. The best results were obtained with dichloromethane (Figure 2). This can be related to the polarity of these compounds. Furthermore, a protein precipitation method using methanol and acetonitrile as polar solvents was tried and showed very low extraction recovery as well as high matrix effects. The selection of ketotifen as an IS was based on its physicochemical properties [18], which are similar to those of SR and thus generated comparable retention times. Moreover, this selection satisfied several other criteria including stability, purity and compatibility with the detector. 

**Figure 3:**
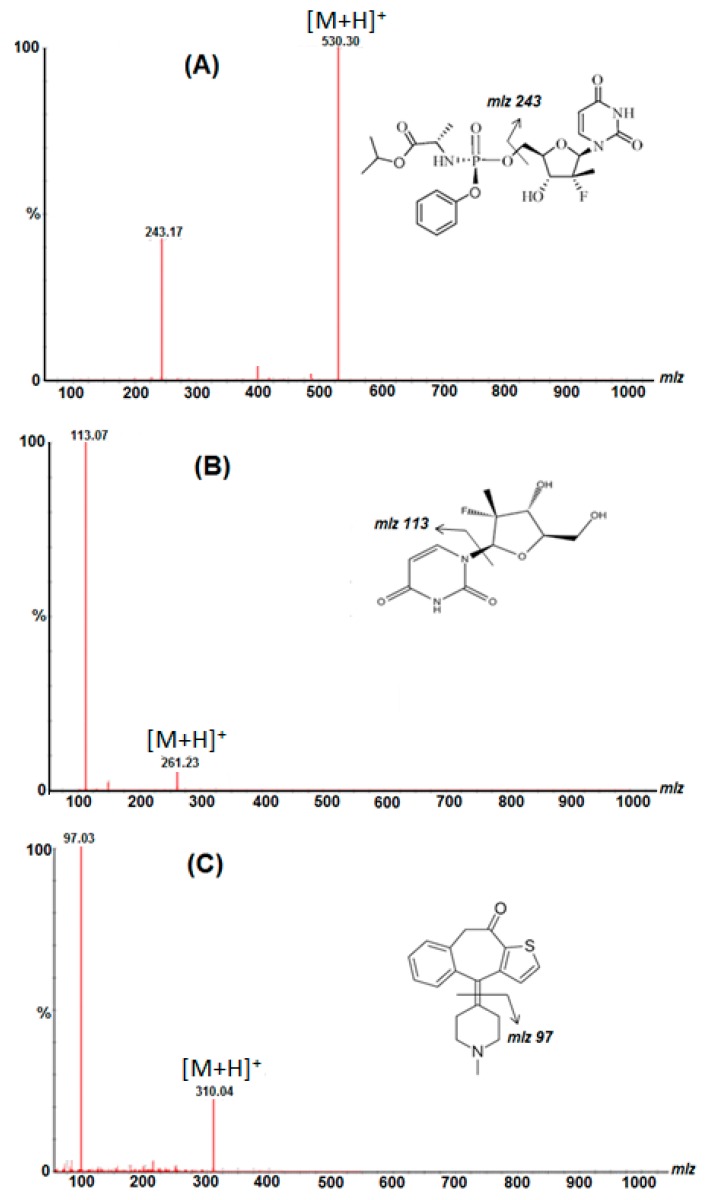
Structures, suggested fragmentation patterns and full-scan product ion spectra of sofosbuvir (**A**)**,** GS331007 (**B**) and ketotifen IS (**C**).

Negative and positive ionization modes were also used for the detection of SR and its metabolite GS331007, showing that the positive ionization was more sensitive than the negative mode. The detection of compounds of interest was achieved using MRM to obtain the maximum detection sensitivity and selectivity as mentioned under the experimental section. The mass spectrometry (MS1) scan in the positive mode of SR, GS331007 and IS showed the main protonated precursor [M + H]^+^ ions at *m*/*z* 530.3, 261.26 and 310.20, respectively. Alongside this, the detection of the fragments was achieved from the daughter ion scan of the obtained precursor ions, as illustrated in Figure 3. 

In order to achieve the best peak symmetry and intensity, several chromatographic conditions were tried including the variation of stationary as well as mobile phases; initially, we tried formic acid 0.1% in an aqueous solution with different ratios of methanol and acetonitrile separately as organic modifiers. It was found that acetonitrile provides better peak sensitivity than methanol (Supplementary Materials Figures S1 and S2). Acetonitrile as the organic modifier was then used with different buffer solutions such as ammonium acetate, ammonium formate and ammonium formate with the addition of 0.1% formic acid. The optimum peak sensitivity and symmetry were obtained using acetonitrile:5 mM ammonium formate:0.1% formic acid (85:15:0.1% *v*/*v*/*v*) as a mobile phase at a flow rate of 0.35 mL/min in an isocratic elution, and the separation was achieved on Acquity UPLC BEH C_18_ (50 × 2.1 mm, i.d. 1.7 µm, Waters, USA). SR, GS331007 and IS were eluted in a very short retention time—equivalent to 1.83, 1.61 and 1.35 min—which is advantageous for high-throughput screening in routine analysis and bioequivalence studies in human samples. Although SR, GS331007 and IS have very similar retention times, the accuracy and precision of the results remained unaffected because quantification was performed using multiple-reaction monitoring (MRM).

## 3. Discussion

### 3.1. Method Validation

#### 3.1.1. Selectivity

**Figure 4:**
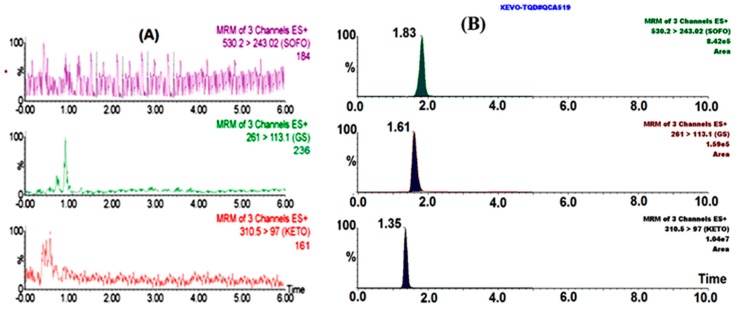
Chromatogram of (**A**) drug-free human plasma, (**B**) spiked plasma with sofosbuvir, GC331007 and ketotifen (IS) from top to bottom.

The method selectivity was assessed by analyzing replicates of plasma samples spiked with the LLOQ of SR, its metabolite GS331007 and IS, and comparing them with those of blank plasma. The obtained chromatograms show that there are no secondary peaks or endogenous components interfere with the analyte peaks under the established chromatographic conditions. These results indicate that the proposed analytical method is highly selective toward the determination of SR and its metabolite GS331007 in human plasma, as illustrated in Figure 4.

#### 3.1.2. Linearity and Limit of Quantification

The linearity of the proposed methods was determined by plotting the peak area ratio of SR and its metabolite GC331007 to IS versus the relative concentrations using the linear least square regression method. A linear response was observed over the examined concentration range (2–1000 ng/mL) for SR and from 10–1500 ng/mL for its metabolite GC331007. The generated calibration curves suggest that the linearity of the method was good. The limit of detection (LOD) and limit of quantification (LOQ) were calculated based on the SD of the response and slope (S) using the following equations: LOD = 3.3 SD/S, LOQ = 10 SD/S, and the limit of quantitation was 1 ng/mL for SR and 10 ng/mL for GS331007. The limit of detection was 0.35 ng/mL for SR and 3 ng/mL for GS331007, as shown in Table 1.

#### 3.1.3. Precision and Accuracy

The precision of intra- and inter-day results of targeted analytes (expressed as % RSD) in the quality control (QC) samples were found to be in the ranges of 3.88–6.14% and 3.57–6.80%, respectively. Accuracy—which is defined as the closeness between the true and the experimental values and expressed as mean recovery % of intra-day and inter-day results for the studied QC samples—was in the ranges of 98.05–112.94% and 98.41–103.05% for SR and GS331007, respectively. These results indicate that the proposed method is precise, accurate and that the assay values satisfy the acceptance criteria (±15%) for both precision and accuracy. Table 2 summarizes the results.

#### 3.1.4. Extraction Recovery

The percentage recoveries (mean ± SD) of SR and its metabolite GS331007 obtained from plasma at three different QC levels were 88.6 to 95.2% and 86.3% to 89.1%, respectively. The mean extraction recovery for ketotifen IS was calculated to be 92.7%. The obtained results show that the extraction method of SR and its metabolite GS331007 was efficient and concentration-independent.

#### 3.1.5. Stability Experiments

All stability tests for SR and its metabolite GS331007 are summarized in Table 3. The stabilities of SR and its metabolite GS331007 solutions were analyzed at two different concentrations of the QC samples (low and high). The RSD% of the obtained results were within ±6.7% (less than ±15), indicating that SR and its metabolite spiked in plasma were stable and did not show a significant decomposition under the investigated storage conditions. Furthermore, the standard solutions of SR, GS331007 and IS were found to be stable for a period of 15 days at refrigerator temperature (below 8 °C). Moreover, the stability results showed that SR and the metabolite were stable for a period of 30 days at −80 °C in spiked plasma and for more than 20 days in an aqueous solution kept in a refrigerator, as described previously in Section 4.5.6. 

#### 3.1.6. Matrix Effect

The effect of plasma endogenous components on the determination of analytes in human plasma was evaluated by comparing the peak area from plasma samples spiked with SR and its metabolite on three different concentration levels, as described in Table 4, with the response of analytes of blank samples at identical concentrations. The obtained results of the matrix effects on the compounds under investigation showed that recovery values ranged from 95.4% to 97.2%, with a negligible matrix effect (3%). Moreover, the matrix effects of the IS were also negligible (2%). These results indicate that the plasma components have negligible effects on the ionization of analyte under the optimized conditions. 

## 4. Experiment

### 4.1. Chemicals 

High purity (>99%) SR, GS331007 and ketotifen were purchased from Sigma Aldrich (St. Louis, MO, USA). 

All aqueous solutions were prepared using LC-MS grade water (Sigma Aldrich, Darmstadt, Germany). The acetonitrile and methanol used in this study were LC-MS grade (Sigma Aldrich, Darmstadt, Germany). Ammonium formate, dichloromethane, ethyl acetate, hexane, diethyl ether and formic acid were purchased from Sigma Aldrich (Darmstadt, Germany).

Blank plasma was obtained from the University of Sharjah Hospital, United Arab Emirates (UAE), and was stored at −80 °C.

### 4.2. Instrumentation 

The separation and mass spectrometric analysis of the target compounds was achieved on a Waters Acquity UPLC H-Class-Xevo Triple Quadrupole system (Milford, MA, USA) coupled with an electrospray ionization source (ESI). The chromatographic separation was achieved using a mixture of acetonitrile:5 mM ammonium formate:0.1% formic acid (85:15:0.1% *v*/*v*/*v*) as a mobile phase at a flow rate of 0.35 mL/min in an isocratic mode, and the overall run time was 2.0 min. The column temperature was kept at 25 °C and the pressure of the system was maintained at 6000 psi. The optimized parameters were maintained for both the analyte and the IS as follows: cone gas flow, 6 L/hour; nitrogen gas flow, 600 L/hour; capillary voltage, 2.3 kV; the ion source temperature, 150 °C; and the desolvation temperature was set at 300 °C. The compound parameters such as cone voltage and collision energy were optimized and set at 30 V and 21 eV for SR; 30 V and 15 eV for GS331007; 30 V and 24 eV for IS. Quantification was performed using MRM mode by monitoring the parent and daughter ions of *m*/*z* 530.20 to m/z 243.02 for SR, *m*/*z* 261.00 to *m*/*z* 113.10 for GS331007 and *m*/*z* 310.50 to *m*/*z* 97.00 for IS. 

### 4.3. Calibration Curve and Quality Control Samples

Standard stock solutions (1 mg/mL) of SR, GS331007 and ketotifen (IS) were prepared separately by dissolving the required amounts in methanol. Working stock solutions of 2 µg/mL were then prepared by the dilution of stock solutions and kept at −20 °C. Seven solutions at seven different concentrations of combined solution of SR and its metabolite were prepared daily using the proper dilution of the working stock solutions to prepare calibration curve and quality control (QC) samples. Fresh standard solutions and the QC samples were constituted by spiking 25 µL of the combined solution in addition to 25 µL of IS (2 µg/mL) to 450 µL of control human plasma as shown in Table 5.

### 4.4. Preparation of Samples

A volume of 450 µL of plasma was spiked with 25 µL of the proper concentration of SR, GC331007 and 25 µL of ketotifen (the final concentration of IS was 100 ng/mL). Then 3000 µL of dichloromethane was added to the spiked plasma for LLE. The solution was vortexed for 1 min and centrifuged for 10 min at 3500 rpm to permit the separation of the two layers. A 2.5 mL sample of the organic phase was transferred to another dry tube and a stream of nitrogen gas was applied to completely dry the organic solvent. The residue was completely dissolved in 500 µL of methanol and a volume of 10 µL of this solution was then analyzed using the UPLC-MS/MS system.

The unknown concentrations of SR and GS331007 samples were calculated using the established calibration curves.

### 4.5. Method Validation

The optimized LC-MS/MS was validated for its linearity, sensitivity, accuracy, precision, specificity and matrix effect, following the bioanalytical method validation guidelines proposed by the Food and Drug Administration (FDA) [19].

#### 4.5.1. Specificity and Selectivity 

Six different plasma batches were analyzed to verify the absence of endogenous components that might interfere with the analyte peaks. 

#### 4.5.2. Calibration Curve

Seven different working standard solutions of SR and GC331007—equivalent to 2, 10, 20, 100, 500, 750 and 1000 ng/mL for SR, and 10, 20, 100, 500, 750, 1000 and 1500 ng/mL for GC331007—were prepared to evaluate the linearity of the optimized method. Calibration curves were obtained by plotting the peak area of the analytes to that of the IS against the relative standard concentrations. The low quality control (LQC), medium quality control (MQC) and high quality control (HQC) for SR were 15, 300 and 850 ng/mL, and for GC331007 were 15, 300 and 1250 ng/mL. Plasma blank samples (plasma processed without IS addition) and zero blank samples (plasma processed with the IS addition) were injected with each calibration curve. The standard deviation (SD) of the obtained results should not exceed ± 15% of nominal concentrations, except for the LLOQ, where the acceptance criteria is ±20% [19]. 

#### 4.5.3. Precision and Accuracy 

The evaluation of both inter- and intra-day precision and accuracy was performed by injecting 18 replicates of three different quality control solutions as mentioned in Table 3, on the same and different days. The acceptance criteria for accuracy is ± 15% expressed as SD from the true value and the acceptance criteria for the precision should be equal or less than 15% expressed as relative standard deviation (RSD%).

#### 4.5.4. Recovery

The extraction recovery of SR, GS331007 and IS was evaluated by comparing the peak areas of three QC levels (LQC, MQC and HQC) of standard solutions to the response area of spiked plasma with the same concentrations [13].

#### 4.5.5. Matrix Effect

The matrix effect of plasma elements on analyte ionization was evaluated by the comparison of peak areas for plasma samples spiked with SR, GS331007 and IS peaks, with the peak areas of analytes in blank samples at identical concentrations [20,21].

#### 4.5.6. Stability 

The stability of SR, GS331007 and IS of two different QC levels equivalent to 15 and 850 ng/mL for SR, and 15 and 1250 ng/mL for GS331007 in human plasma, was evaluated by analyzing six replicates of each sample. The samples were kept in different storage conditions. In the bench top condition, the samples were kept at room temperature for 6 h. While in freeze/thaw stability, the samples were frozen at −20 °C and thawed for three successive cycles. For the long-term stability, the samples were analyzed after being kept at −80 °C for period of 30 days. In the auto sampler stability, the samples were stored after solvent extraction in an auto sampler for 24 h at 4 °C then analyzed. All the peak areas of the injected solutions were compared to the freshly prepared calibration curve. Samples were considered stable if the % recovery of the injected solutions were within the acceptance criteria of ±15% of the nominal concentration. 

## 5. Conclusions

A rapid and sensitive UPLC-ESI-MS/MS method was developed and validated for the simultaneous estimation of SR and its metabolite in human plasma. The method was fully validated according to the bioanalytical method validation guidelines. The method was found to be precise, accurate and specific; furthermore, the proposed method shows significant advantages to previously published methods in term of sensitivity and analysis time. The obtained results of the matrix effects indicate that ion suppression or enhancement from human plasma components was negligible under the optimized conditions. The proposed method can be therefore applied in the high-throughput analysis required for pharmacokinetic and bioequivalence studies in humans.

## Figures and Tables

**Table 1:** The linearity and lower limit of quantification (LLOQ) of sofosbuvir and GS331007.

Compound	Linear Range (ng/mL)	Linear Equation (n = 6)	Correlation Coefficient	Limit of Quantification (LOQ) (ng/mL)	Limit of Detection (LOD) (ng/mL)
SR	1–1000	y = 0.0009222x + 0.001769 ^a^	0.9972	1	0.35
GS331007	10–1500	y = 0.0000378x + 0.001339 ^b^	0.9972	10	3

^a^ For SR, the SD of the slope is 0.00012, and the SD of the intercept is 0.00082; ^b^ For GS331007, the SD of the slope is 0.00000118, and the SD of the intercept is 0.00066.

**Table 2:** Intra- and inter-day accuracies and precisions of sofosbuvir and GS331007 in plasma.

Analyte		(Inter-Day Assay) (n = 18)			(Intra-Day Assay) (n = 6, for 3 Days)		
SR	Nominal conc. (ng/mL)	15	300	850	15	300	850
	Conc. (ng/mL)	15.7	332.0	959.9	15.8	294.0	926.0
	RSD (%)	5.79	5.99	3.99	5.93	3.88	6.14
	Mean recovery %	104.89	110.67	112.94	105.17	98.04	108.96
GS331007	Nominal conc. (ng/mL)	15	300	1250	15	300	1250
	Conc. (ng/mL)	14.8	299.4	1230.1	14.9	309.1	1286.4
	RSD (%)	5.54	6.80	3.90	5.68	4.95	3.57
	Mean recovery %	98.89	99.80	98.41	99.67	103.05	102.92

**Table 3:** Stability of SR and GS331007 in a matrix using the proposed analytical method.

Analyte	SR (n = 6)		GS331007 (n = 6)	
Stability Conditions	15 (ng/mL)	850 (ng/mL)	15 (ng/mL)	1250 (ng/mL)
Post preparative stability at 4 °C				
Concentration (ng/mL)	16.2	898.8	15.7	1362.4
Mean recovery % ± RSD	108 ± 5.7	105.74 ± 4.6	105 ± 3.0	109 ± 0.6
Bench top (6 h)				
Concentration (ng/mL)	16.8	918.0	15.5	1346.3
Mean recovery % ± RSD	112.2 ± 1.9	108.0 ± 5.4	103.3 ± 5.9	107.7 ± 3.0
Freeze thaw (three cycles)				
Concentration (ng/mL)	16.3	911.1	16.8	1344.0
Mean recovery % ± RSD	108.6 ± 2.8	107.1 ± 6.7	112.0 ± 3.1	107.52 ± 4.1
30 days at −80 °C				
Concentration (ng/mL)	16.2	977.0	2.5	1361.3
Mean recovery % ± RSD	108.2 ± 2.5	114.9 ± 1.3	106.2 ± 1.9	108.9 ± 1.5

**Table 4:** Mean recovery (n = 5) and matrix effect (ME) for SR and GS331007 at two different concentration levels.

Analyte	Spiked Conc. ng/L	Mean Recovery %	Recovery RSD	Mean ME %
SR	15	97.2	6.3	3
	850	95.4	4.7	2
GS331007	15	99.1	4.8	2
	1250	96.6	3.9	1

**Table 5:** Standard curve concentrations and quality control samples for sofosbuvir (SR) and its metabolite, GS331007 (LQC: low conc. quality control, MQC: medium conc. quality control, HQC: high conc. quality control).

Prepared Samples	Plasma Volume	Adding 25 µL of Each Working Standard Solution (ng/mL)		Final Volume	Final Plasma Concentration (ng/mL)	
		SR	GS331007		SR	GS331007
Calibrators	450 µL	40	200	500 µL	2	10
		200	400		10	20
		400	2000		20	100
		2000	10,000		100	500
		10,000	15,000		500	750
		15,000	20,000		750	1000
		20,000	30,000		1000	15,000
LQC	450 µL	300	300	500 µL	15	15
MQC		6000	6000		300	300
HQC		17,000	25,000		850	1250

## Supplementary Materials

The following are available online at https://www.mdpi.com/1420-3049/24/7/1302/s1, Figure S1: The effect of ammonium acetate on peak shape and symmetry, Figure S2: Methanol effect on the retention time.
