# Supplementary material for: Quick and Sensitive UPLC-ESI-MS/MS Method for Simultaneous Estimation of Sofosbuvir and Its Metabolite in Human Plasma
Source: Molecules. 2019 Apr 3;24(7):1302. doi: 10.3390/molecules24071302 (PMC6480583; doi:10.3390/molecules24071302)
Supplement: Supplementary file 1 [file molecules-24-01302-s001.pdf]

Supplementary materials

Quick and Sensitive UPLC-ESI-MS/MS Method for Simultaneous Estimation of Sofosbuvir and Its Metabolite in Human Plasma

Mohammad H. Semreen [1,2,*], Hasan Y. Alniss [1,2], Muath K. Mousa [3] and Hassan Y. Aboul-Enein [4]

1 College of Pharmacy, University of Sharjah, P.O. Box 27272, Sharjah, Saudi Arabia; halniss@sharjah.ac.ae
2 Sharjah Institute for Medical Research, University of Sharjah, P.O. Box 27272, Sharjah, Saudi Arabia
3 Research Institute of Science and Engineering, University of Sharjah, P.O. Box 27272, Sharjah, Saudi Arabia; mmousa2@sharjah.ac.ae
4 Pharmaceutical and Medicinal Chemistry Department, Pharmaceutical and Drug Industries Research Division, National Research Centre, P.O. Box 12622, Dokki, Giza, Egypt; haboulenein@yahoo.com
* Correspondence: msemreen@sharjah.ac.ae; Tel.: +97165057419

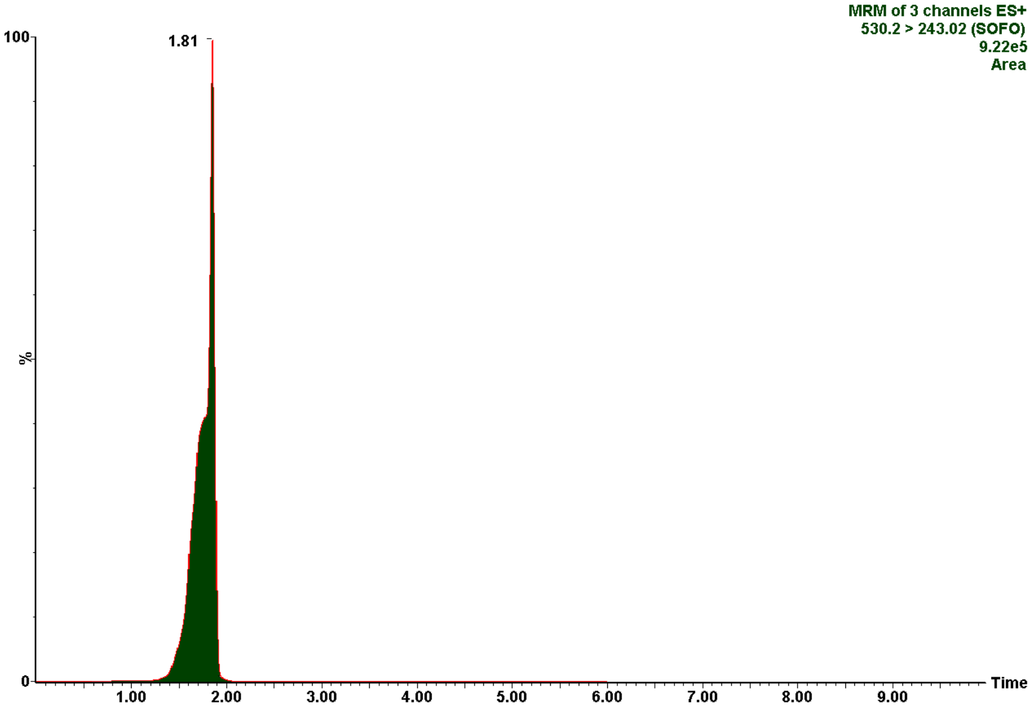

S1: The effect of ammonium acetate on peak shape and symmetry.

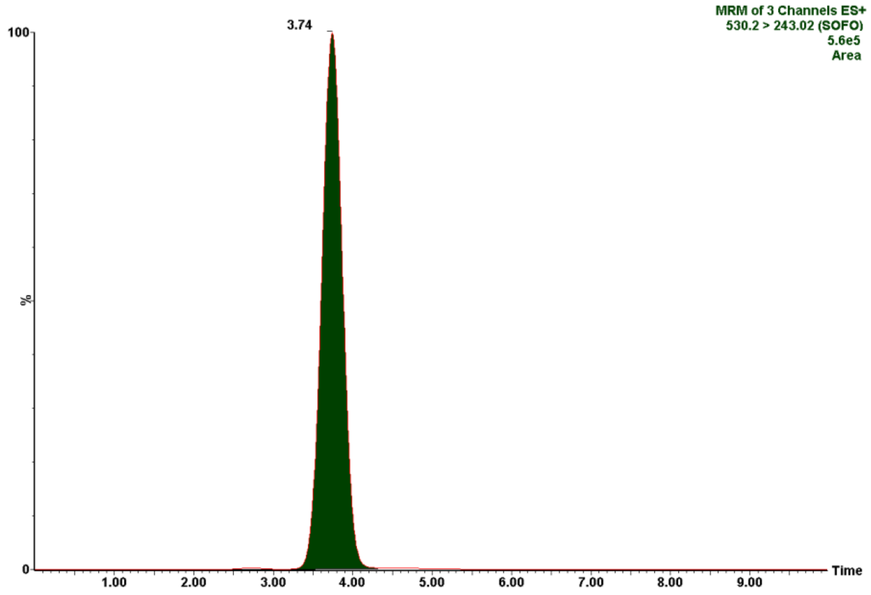

S2: Methanol effect on the retention time (late elution).
